# University of Buffalo

**Published:** 1903-06

**Authors:** 


### University of Buffalo.

THE fifty-seventh annual commencement of the department of medicine, U. of B., was held on Tuesday, May 6, 1903. The graduating exercises were held in the Teck Theater at 11 o'clock a.m., and were opened with prayer by the Rev. J. A. Regester of St. Paul's Cathedral. Dr. John Parmenter, secretary of the faculty, presented the names of the candidates to the chancellor and Dr. Matthew D. Mann, dean of the faculty, administered the hippocratic oath. The degree of doctor of medicine was conferred by the Hon. Wilson S. Bissell, chancellor of the University, upon the following-named candidates:

Clarence Beals, John Lewis Bishop, Bert John Bixby, Charles Lisle Bond, Elliott T. Bush, William Henry Callahan, Frank Oliver Cole, John A. Conway, Carlos Emmons Cummings, A. B.; Spencer Arthur Drake, Edward Henry Drozeski, Clarence Christopher Duchscherer, James E. Eisenhart, Ph. M.; George Louis Fischer, David Earl Fraser, May Gibson, Edward Eugene Gillick, Walter Speisser Goodale, James McAndrew Happell, Glenn H. Hardy, Albert Jabesh Harris, B.S.; Lawrence Aloysius Highland, A.B.; Frank Jones, Leon M. Kysor, James Russell Lowell, John Wesley Munro, Albert William Palmer, Frederick James Parmenter, Fred Cole Purcell, Edwin David Putnam, Hyatt Regester, Edwin Albert Riesenfeld, Carroll Julian Roberts, Hibbert Rice Roberts, Edward Wesley Roos, Burton Thorne Simpson, Edward Hugo Storck, Chris Lester Suess, George Calvin Swerdfeger, Willard Hall Veeder, L. Edward Villiaume, John Lewis Washburn, Ph.C.; Harry Milton Weed, Thew Wright, A.B.; Shin Yokoyama, M.B.

The sixteenth annual commencement of the department of pharmacy and the eleventh annual commencement of the department of dentistry, were held at the same time and under similar ceremonies. The former class numbered 24 and the latter 68.

The honor members in the class of medicine were as follows:

Hyatt Regester, Leon M. Kysor, Elliott T. Bush, Thew Wright, A.B.; Harry Milton Weed, Burton Thorne Simpson,

Chris Lester Suess, Edwin David Putnam, Bert John Bixby, Edward Henry Drozeski, Frederick James Parmenter, Carlos Emmons Cummings, A.B.; John Lewis Washburn, Ph.C.

The Reverend R. R. Converse, D.D., of Rochester, delivered the address to the graduates.

The exercises were interspersed with musical selections and closed by a benediction.

ALUMNI ASSOCIATION.

The twenty-eighth annual meeting of the alumni association of the medical department was held on commencement day. In the morning the classes of '53, '63, '73, '83, and '93 held reunions.

The Alumni Association met in the university building in the afternoon. Professor William T. Sedgewick of the Massachusetts Institute of Technology was the principal speaker. His subject was: The protection of public health by filtration of municipal water supplies. His address was discussed by Colonel Thomas W. Symons, Dr. W. D. Greene, Dr. Herbert M. Hill, Dr. Herbert U. Williams, Dr. William R. Campbell, Dr. Arthur W. Henchell, and Dr. George Fell.

The Alumni Association elected these officers: president, A. W. Henchell, '89, Rochester; first vice-president, Fridolin Thoma, '87, Buffalo; second vice-president, Henry T. Benham, '90, Honeoye Falls; third vice-president, Jane W. Carroll, '91, Buffalo; fourth vice-president, George A. Himmelsbach, '91, Buffalo; fifth vice-president, C. J. Reynolds, '90, Buffalo; secretary, Thomas H. McKee, '97, Buffalo; treasurer, Herman K. DeGroat, '97, Buffalo. Board of trustees, D. A. Currie, '64, 1903, Englewood, N. J.; A. W. Bayliss, '89, Buffalo; William Warren Potter, '59, 1905, Buffalo; D. W. Harrington, '71, 1906, Buffalo; F. H. Moyer, '72, 1907, Moscow. Executive committee, Albert T. Lytle, '93, chairman, Buffalo; Grover W. Wende, '89, Buffalo; Franklin W. Barrows, '93, Buffalo; A. W. Henchell, *ex-officio*, Rochester; John Parmenter, *ex-officio*, Buffalo.

